# Genetic susceptibility, inflammation and specific types of depressive symptoms: evidence from the English Longitudinal Study of Ageing

**DOI:** 10.1038/s41398-020-0815-9

**Published:** 2020-05-12

**Authors:** Philipp Frank, Olesya Ajnakina, Andrew Steptoe, Dorina Cadar

**Affiliations:** 1 Department of Behavioural Science and Health, University College London, 1-19 Torrington Place, London, WC1E 7HB UK; 2 Department of Biostatistics & Health Informatics, Institute of Psychiatry, Psychology and Neuroscience, King’s College London, 16 De Crespigny Park, London, WC1E 7HB UK

**Keywords:** Psychology, Genetics, Depression, Predictive markers

## Abstract

Genetic susceptibility to depression has been established using polygenic scores, but the underlying mechanisms and the potentially differential effects of polygenic scores on specific types of depressive symptoms remain unknown. This study examined whether systemic low-grade inflammation mediated the association between polygenic scores for depressive symptomatology (DS-PGS) and subsequent somatic versus cognitive-affective depressive symptoms. The sample consisted of 3510 men and women (aged 50+) recruited from the English Longitudinal Study of Ageing. DS-PGS were derived using the results of a recent genome-wide association study. Plasma C-reactive protein (CRP) was measured at wave 6 (2012/13). Depressive symptoms were assessed at wave 8 (2016/17), using the eight-item version of the Centre for Epidemiological Studies Depression Scale. Covariates (wave 2, 2004/05) included age, sex and ten principal components (PCs) to control for population stratification. Confirmatory factor analysis was performed to corroborate a previously identified two-factor structure of the CES-D, distinguishing between cognitive-affective and somatic symptoms. Longitudinal structural equation modelling was used to investigate the mediating role of CRP in the relationship between DS-PGS and cognitive-affective versus somatic symptoms. Our results showed that participants with a higher polygenic susceptibility to DS were significantly more likely to report cognitive-affective and somatic symptoms at follow-up. Mediation analyses revealed that CRP mediated the relationship between DS-PGS and somatic symptoms, but not the association between DS-PGS and cognitive-affective symptoms. These differential effects highlight the importance of considering individual differences in depression profiles in future studies. Ultimately, this will inform healthcare professionals to design more targeted treatments.

## Introduction

Depression is a leading cause of disability and a growing public health concern worldwide, particularly among older adults^1,2^. It is typically characterised by a number of cognitive, affective and somatic symptoms, including anhedonia, low mood, loss of appetite, and sleep problems^3^. According to a previous prevalence study, ~18% of adults aged 65 years and older in England experience elevated depressive symptoms^4^. Research also indicates that depression has substantial public health implications. For example, depression has been linked to an increased risk of morbidity^5–7^, cognitive decline^8^, and mortality^9,10^. In the past decades, research has attempted to uncover plausible biopsychosocial pathways contributing to the development of depression. However, the exact biological, psychological and social mechanisms underlying the pathogenesis of depression remain elusive.

Genetic variation may play an important role in the aetiology of depression. The advent of genome-wide association studies (GWAS) has made it possible to investigate the genetic underpinnings of complex psychiatric disorders, including depression^11^. GWAS aim to identify individual genetic variants, also known as single nucleotide polymorphisms (SNPs), which are significantly associated with a trait or phenotype of interest^12^. However, for many complex psychiatric disorders, individual SNPs have been found to exert only modest effects on these conditions, making it difficult to predict disease risk reliably^13^. Recent evidence suggests that depression is likely to have a substantial polygenic component^14^. Accordingly, genetic susceptibility to depression cannot be simply explained by the independent contributions of individual genetic variants, but rather by the combined additive effects of multiple SNPs in the human genome^15^. This has led to the development of polygenic scores (PGS), which summarise genome-wide genotyping data into a single score that measures genetic susceptibility to a particular trait or illness. A number of previous studies have examined the associations between PGS for depression and depressive symptoms^14,16–18^. Interestingly, PGS have been found to significantly correlate with disease status and chronicity of the disorder^16,19^. Moreover, they typically explained 0.2–1% of variance in depression, suggesting that other factors contribute to the pathogenesis of depression^19,20^.

An abundance of studies has indicated a link between peripherally and centrally localised inflammatory activity and the pathogenesis of depression^21–23^. Considerable evidence suggests that systemic pro-inflammatory cytokines (i.e., chemical messengers of the immune system) are capable of signalling the central nervous system to alter behaviour and mood states^24^. This is consistent with the cytokine hypothesis of depression, which posits that systemic low-grade inflammation and subsequent neuroendocrine responses can induce depressive-like symptoms in a subset of people^25^. Systemic low-grade inflammation describes the persistent production of pro-inflammatory factors in response to immune challenge and stress, which may result in a prolonged state of low-grade inflammation^26^. It is typically measured via serum concentrations of circulating pro-inflammatory markers, including the cytokines interleukin-6 (IL-6) and interleukin-8 (IL-8), the acute phase protein C-reactive protein (CRP), the coagulation protein fibrinogen or the tumour necrosis factor α (TNF-α). Plausible biological pathways exist for the effects of systemic low-grade inflammation on mood. These include the cytokine-induced activation of afferent fibres of the vagus nerve and the transportation of cytokines through the blood-brain barrier, initiating a constellation of depressive-like symptoms, collectively known as ‘sickness behaviour’^24,27^. Sickness behaviour usually refers to a set of primarily somatic symptoms, such as fatigue, sleep problems, reduced locomotor activity and loss of appetite^27^. Notably, most evidence supporting the inflammation hypothesis of depression has been cross-sectional, and prospective studies have found mixed results^28,29^. This could be attributed to the possibility of symptom-specific associations between systemic low-grade inflammation and depression, owing to the prevailing use of sum scores rather than factor analytic symptom structures or individual symptoms of depression^30^. Depression is a multifaceted disorder with varying types of symptom expressions. Previous factor analytic studies suggest a plausible two-factor structure of depression, distinguishing between a cognitive-affective and somatic symptom dimension of depression^31^.

Taken together, evidence suggests that polygenic susceptibility to depression is associated with an increased risk of developing future depressive symptoms. In addition, inflammatory processes have been identified to be another, potentially aetiological, risk factor for DS. However, it remains unclear whether people with increased genetic susceptibility to depression may also be more likely to show elevated levels of systemic low-grade inflammation. Furthermore, there have been no prospective studies investigating whether systemic low-grade inflammation mediates the relationship between PGS for depressive symptomatology (DS-PGS) and specific symptom dimensions of depression. Therefore, the aim of the present was first, to investigate the association between a GWAS-derived DS-PGS and symptom-specific dimensions of depression (i.e., cognitive-affective versus somatic symptoms) in a sample of English community-dwelling older adults. Second, we examined whether systemic low-grade inflammation acted as a downstream mediator in the association between DS-PGS and self-reported cognitive-affective versus somatic symptoms.

## Method

### Study design and participants

The English Longitudinal Study of Ageing (ELSA) is an ongoing, nationally representative, longitudinal population study of adults aged ≥50 years living in England. ELSA was established in 2002, using a variety of biological, psychological and sociological measures to assess the causes and consequences of health-related outcomes. Further details on ELSA have been described in-depth elsewhere^32^. Participants have been followed up bi-annually from 2002 onwards. So far, there have been 8 waves of data collection. ELSA provides a rich variety of psychosocial data collected via face-to-face computer-assisted personal interviews and self-completion questionnaires. Biological data (e.g., blood samples, body mass index, etc.) was collected during nurse visits at participants’ homes. Genome-wide genotyping was conducted at the University College London Genomics. The follow-up period reported here is wave 8 (2016/17), the latest available wave of data collection in ELSA. A total of 8780 participants attended the core assessment at wave 2. Genotype data on depressive symptoms were available for 7183 participants. Participants aged <50 years (*N* = 51) and those with missing blood samples (*N* = 3419) were excluded from the analytical sample. Missing biological data were mainly due to participants’ unwillingness to consent or ineligibility to provide blood samples (e.g., due to clotting or bleeding disorders, use of anticoagulant medication or a history of convulsions). Study participants with CRP levels ≥10 mg/L (wave 6) were also excluded from the analysis (*N* = 212), since these values may reflect acute inflammatory processes rather than systemic low-grade inflammation^33^. Thus, the final analytical sample consisted of 3510 participants. All participants provided informed consent prior to their participation in the study. Ethical approval was obtained from the London Multi-Centre Research Ethics Committee (https://www.elsa-project.ac.uk).

### Measures

#### Outcome: depressive symptoms at wave 8 (2016/17)

Depressive symptoms were measured using the eight-item version of the Centre for Epidemiological Studies Depression Scale (CES-D)^34^. Participants were asked to indicate whether they had experienced low energy levels, effort doing things, low mood, enjoyment/happiness in life, sadness, loneliness and/or sleep problems during the past week. The CES-D uses a binary response scale (yes = 1*;* no = 0). Scores ranged from 0 (i.e., no depressive symptoms) to 8, with scores equal to or higher than three corresponding to the CES-D 20 cut-off of 16 points—representing a clinical diagnosis of depression^35^. The 8-item version of the CES-D is a well-validated screening tool for DS^36^ and has been used often in previous longitudinal investigations^37,38^.

#### Exposure: polygenic scores for depressive symptoms

ELSA participants of European ancestry were genotyped in 2013/14, using the Illumina HumanOmni2.5 BeadChips (HumanOmni2.5-4v1, HumanOmni2.5-8v1.3), with a coverage of ~2.5 million SNPs that capture the genomic variation down to 2.5% minor allele frequency. A full quality control protocol has been described elsewhere (https://www.elsa-project.ac.uk/genetics). Briefly, individuals with suspected non-European ancestry and heterozygosity scores >3 standard deviations from the mean were removed. Furthermore, initial quality control measures were conducted to test for duplicates and missingness of more than 2% of the genotype data. SNPs with a call rate of <98%, a minor allele frequency of <0.01%, and Hardy-Weinberg Equilibrium *p* values of <10^−4^ were excluded. Non-autosomal markers were also removed, as well as regions known to contain clusters of highly correlated SNPs, as these can bias the analyses^39,40^. Principal components analysis was performed to investigate population structure, and ten PCs were retained to account for any ancestry differences in genetic structures^41^. A total of 7183 samples and 1,372,240 genetic variants remained after quality control. DS-PGS calculation was informed by the GWAS summary statistics from the Social Science Genetic Association Consortium (SSGAC)^42^. The SSGAC GWAS meta-analysed the results from a mega-analysis conducted by the Psychiatric Genomics Consortium (PGC)^19^, and the results from two more recent investigations using data from UK Biobank (UKB)^43^ and the Resource for Genetic Epidemiology Research on Aging (GERA) Cohort (*N* = 180 866) (https://www.ncbi.nlm.nih.gov/projects/gap/cgi-bin/study.cgi?study_id=phs000674.v1.p1). In UKB, a continuous phenotype measure was derived, based on two questions asking participants about the frequency they experienced feelings of unethusiasm/disinterest and depression/hopelessness. PGC and GERA used case-control data on major depressive disorder. In addition, SSGAC performed a comprehensive replication analysis with data from 23andMe (*N* = 368,890). DS-PGS were computed using methods previously described in the Health and Retirement Study^44^. Accordingly, DS-PGS were calculated as the sum of SNPs associated with depressive symptoms, weighted by their corresponding effect sizes from the SSGAC summary statistics^45^. PGS analyses were performed based on a recommended *p* value threshold of 1^44^, using the statistical software packages PRSice^46^ and PLINK version 1.9^47^.

#### Mediator: C-reactive protein at wave 6 (2012/13)

Serum concentrations of high-sensitivity CRP (mg/L) were analysed at the Royal Victoria Infirmary laboratory in Newcastle (UK), using the N Latex CRP mono Immunoassay on the Behring Nephelometer II Analyser^48^. CRP values were log-transformed due to their initially skewed distribution.

#### Statistical analysis

Following traditional structural equation modelling (SEM) practice, the analyses were performed in two consecutive steps^49^. First, confirmatory factor analysis (CFA) was conducted to examine the validity of a previously suggested two-factor model of depression^31,50^. The latent construct *cognitive-affective symptom dimension* was hypothesised to represent the common variance among five of the eight CES-D items: ‘did you feel depressed?’,‘did you enjoy life?’, ‘did you feel sad?’, ‘did you feel lonely?’ and ‘were you happy?’. The latent construct somatic symptom dimension was hypothesised to be composed of the remaining three items: ‘did you feel everything you did was an effort?’, ‘was your sleep restless’ and ‘were you unable to get going?’. Second, mediation analysis was performed to examine the standardised direct, indirect and total effect of the association between PGS for DS, systemic low-grade inflammation (i.e., CRP) and the two latent symptom-specific dimensions of the CES-D (i.e., cognitive-affective versus somatic depressive symptoms). Mediation assumes that the relationship between the independent variable and dependent variable is mediated by a third variable. The total effect refers to the effect of DS-PGS on cognitive-affective depressive symptoms and somatic symptoms, respectively. The indirect effect represents the amount of mediation exerted through CRP on the association between the independent and dependent variable. The direct effect describes the effect of the exposure variable on the outcome variable while controlling for the mediator variable^51^. In the current model, the observed cognitive-affective and somatic symptom scores were treated as ordinal variables. Thus, the model was fitted using the robust weighted least squares estimator (WLSMV). This method has been developed specifically for modelling categorical variables. The WLSMV estimator handles missing data by producing unbiased estimates for the parameters and their standard errors. Model fit indices were compared against Pearson *χ*^2^ (Chi-Square) ≤ 0.05, Comparative Fit Index (CFI) ≥ 0.95 and Root Mean Square Error of Approximation (RMSEA) ≤ 0.05. Bootstrapping (1000 replications) was employed as a validated method to estimate the bias-corrected confidence intervals of the indirect effect^52^. All analyses were adjusted for age, sex and ten PCs to account for any ancestry differences in genetic structures that could potentially bias the results.

Descriptive statistics were analysed using the statistical software package STATA version 15. SEM was performed using the Mplus software version 7 (Muthen and Muthen, Los Angeles, CA).

#### Sensitivity analysis

To further explore the temporal precedence in the relationship between PGS, systemic low-grade inflammation and specific types of depressive symptoms, additional analyses were performed to assess the significance of the indirect effect of CRP while adjusting for cognitive-affective and somatic symptoms at wave 6.

## Results

### Sample characteristics

Descriptive statistics of the sample (*N* = 3 510) are provided in Table 1. The average age of participants was 62 years. There was a slightly higher proportion of women (54%) compared with men (46%) in the analytical sample. The mean concentration of CRP (wave 6) was 2.13 mg/L. The percentage of participants with elevated depressive symptoms at wave 8 (mean total CES-D score ≥ 3) was 17%.

**Table 1:** Sample characteristics.

Total sample (*N* = 3 510)	Missing (%)	Mean (SD)	Frequency (%)
Age	−	62.29 ± 7.74	
Sex	−		
Men			1610 (45.87%)
Women			1900 (54.13%)
C-reactive protein (mg/L)	−	2.13 ± 1.91	
Depressive symptoms (CES-8) wave 8			
Overall score	19.54	1.20 (1.71)	
Cognitive-affective score	19.54	0.51 (1.07)	
Somatic score	19.23	0.69 (0.91)	

ELSA, waves 2–8: *B*: regression coefficient. *β*: standardised regression coefficient. Estimator: WLSMV. Model adjusted for age, sex and ten principal components.

*SE* standard error, *CI* confidence interval.

### Confirmatory factor analysis (CFA)

CFA of the items included in the CES-D 8 indicated that the two-factor model differentiating between cognitive-affective and somatic symptoms fit the data better than the one-factor model (Supplementary Fig. S1). Moreover, the two-factor model demonstrated an adequate discriminant validity, based on a correlation between the two latent variables (i.e., cognitive-affective versus somatic symptoms) of less than 0.85^53^.

### Polygenic scores and cognitive-affective versus somatic symptom dimensions

DS-PGS were significantly associated with both the cognitive-affective (*β* = 0.092, 95% CI: 0.048; 0.135) and somatic (*β* = 0.081, 95% CI: 0.039; 0.123) symptoms after controlling for age, sex and 10 PCs (Table 2).

**Table 2:** Associations between polygenic scores and symptom-specific dimensions of depression (wave 8, 2016/17): cognitive-affective factor versus somatic factor (*N* = 3 510).

Cognitive-affective factor					Somatic factor				
*B*	SE	*p* value	95% CI	*β*	*B*	SE	*p* value	95% CI	*β*
0.007	0.002	<0.001	0.004; 0.010	0.092	0.006	0.002	0.002	0.003; 0.009	0.081

Data source: ELSA, waves 2–8: *B*: regression coefficient. *β*: standardised regression coefficient. Estimator: WLSMV. Model adjusted for age, sex and ten principal components.

*SE* standard error, *CI* confidence interval.

### The mediating role of C-reactive protein

**Fig. 1:**
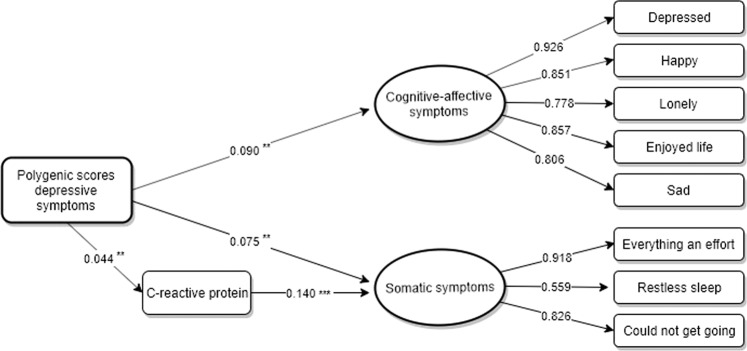
Longitudinal mediation model of the association between polygenic scores for depressive symptomatology, C-reactive protein (wave 6, 2012/13) and specific types of depressive symptoms (wave 8, 2016/17) (*N* = 3510). Data source: ELSA, waves 2–8: Estimator: WLSMV. Model adjusted for age, sex and ten principal. *p* value significance level: *<0.05, **<0.01, ***<0.001.

Mediation analysis was performed to explore whether CRP (wave 6, 2012/13) acted as an intermediate mechanism of the relationship between DS-PGS (wave 2, 2004/05) and cognitive-affective versus somatic symptoms (wave 8, 2016/17). All analyses were adjusted for age, sex and 10 PCs to control for ancestry differences in genetic structures (Table 3). The longitudinal measurement model including DS-PGS, CRP, symptom-specific dimensions of depression and covariates showed a good model fit (RMSA = 0.018, CFI = 0.972, Pearson *χ*^2^ < 0.0001). The results indicated that CRP acted as a partial downstream mediator of the relationship between DS-PGS and somatic symptoms (*β* = 0. 006, 95% CI, 0.002, 0.011), explaining a total of 7.42% of this association. Moreover, a significant direct (*β* = 0. 075, 95% CI, 0.033, 0.117) and total (*β* = 0.081, 95% CI, 0.039, 0.123) effect were found between DS-PGS and the somatic symptom dimension. In contrast, no significant mediating effect of CRP was found for the relationship between DS-PGS and cognitive-affective symptoms (*β* = 0.002, 95% CI, −0.001, 0.004). However, DS-PGS had a significant direct (*β* = 0.90, 95% CI, 0.047, 0.133) effect on cognitive-affective symptoms. Furthermore, there was a significant total effect (*β* = 0.092, 95% CI, 0.048, 0.135) in the association between DS-PGS and cognitive-affective symptoms after adjusting for the mediator variable CRP. Figure 1 provides a graphical illustration of the main results.

**Table 3:** Mediation of the association between polygenic scores for depressive symptomatology and specific types of depressive symptoms (wave 8, 2016/17) through C-reactive protein (wave 6, 2012/13) (*N* = 3 510).

			Total indirect effect	Total effect	Total direct effect	Total effect mediated
Independent variable (wave 2)	Mediator (wave 6)	Outcome (wave 8)	*Standardised Coefficient* (Bc CI)	*Standardised Coefficient* (Bc CI)	*Standardised Coefficient* (Bc CI)	*%*
DS-PGS	C-reactive protein	Cognitive-affective factor	0.002 (−0.001, 0.004)	0.092 (0.048, 0.135)	0.090 (0.047, 0.133)	–
DS-PGS	C-reactive protein	Somatic factor	0.006 (0.002, 0.011)	0.081 (0.039, 0.123)	0.075 (0.033, 0.117)	7.41%

Data source: ELSA, waves 2–8: Bc CI: bias adjusted confidence interval. Estimator: WLSMV. Model adjusted for age, sex and ten principal components to account for ancestry differences.

### Sensitivity analysis

The results of the sensitivity analyses (Table 4 and Fig. S2) revealed that CRP remained a significant mediator in the relationship between PGS and subsequent somatic symptoms of depression after additionally controlling for cognitive-affective and somatic symptoms at wave 6 (*β* = 0.006, 95% CI, 0.002, 0.010). Similar to our main results, no significant indirect effect was found for CRP on the relationship PGS and cognitive-affective depressive symptoms (*β* = 0.002, 95% CI, −0.001, 0.004)

**Table 4:** Mediation of the association between polygenic scores for depressive symptomatology and specific types of depressive symptoms (wave 8, 2016/17) through C-reactive protein (wave 6, 2012/13), additionally adjusted for cognitive-affective and somatic symptoms (wave 6) (N = 3 510).

			Total indirect effect	Total effect	Total direct effect	Total effect mediated
Independent variable (wave 2)	Mediator (wave 6)	Outcome (wave 8)	*Standardised Coefficient* (Bc CI*)	*Standardised Coefficient* (Bc CI)	*Standardised Coefficient* (Bc CI)	*%*
DS-PGS	C-reactive protein	Cognitive-affective factor	0.002 (−0.001, 0.004)	0.093 (0.054, 0.131)	0.091 (0.052, 0.129)	
DS-PGS	C-reactive protein	Somatic factor	0.006 (0.002, 0.010)	0.078 (0.041, 0.115)	0.072 (0.036, 0.109)	7.69%

Data source: ELSA, waves 2–8: Bc CI: bias adjusted confidence interval. Estimator: WLSMV. Model adjusted for age, sex, ten principal components and wave 6 cognitive-affective and somatic symptoms.

## Discussion

This is the first study to investigate the prospective associations between genetic susceptibility to depressive symptomatology, systemic low-grade inflammation (i.e., CRP) and specific types of depressive symptoms (i.e., cognitive-affective and somatic symptoms) in a large nationally representative cohort of older adults. Our findings suggest that increased genetic susceptibility to depressive symptomatology was associated with both subsequent cognitive-affective and somatic depressive symptoms. Moreover, CRP was found to mediate the relationship between DS-PGS and somatic symptoms, but not cognitive-affective symptoms, explaining a total of 7.42% of this association.

The present study moved beyond analysing the effects of single genetic variants and instead examined the cumulative effect of multiple SNPs on subsequent depressive symptoms. The use of genetic data in our analyses provided a measure of depressive symptomatology that preceded both the mediator and the outcome. As such, including DS-PGS implied a clear pathway direction—from genetic polymorphisms to systemic low-grade inflammation and subsequent depressive symptoms.

Our results indicate a relatively small overall effect of DS-PGS on both cognitive-affective and somatic depressive symptoms, respectively. This is in agreement with a number of previous prospective cohort studies suggesting that many loci, each with a modest effect, influence DS^14,16,18,20^. Importantly, previous studies did not take into account symptom-specific effects of PGS. Hence, our findings provide new evidence highlighting the potentially differential effects of DS-PGS on subsequent depressive symptom profiles.

Our mediation analysis revealed that CRP partially mediated the relationship between DS-PGS and subsequent somatic symptoms, but not cognitive-affective symptoms. These associations were independent of age, sex, and 10 PCs. Although the confidence intervals partially overlapped, our findings indicated that, statistically, the results were significantly different for these symptom domains (i.e., latent outcome variables). Further adjustment for cognitive-affective and somatic depressive symptoms measured at the time of the mediator did not significantly change the results. Taken together, our findings support the notion that systemic inflammation may be an inherent mechanism driving the development of somatic depressive symptoms. Specifically, participants with a higher genetic risk for depressive symptoms were significantly more likely to report elevated levels of CRP, which subsequently increased the risk for developing somatic depressive symptoms, but not cognitive-affective symptoms.

To date, prospective evidence supporting the depressogenic effects of systemic low-grade inflammation has yielded mixed results. For instance, a previous meta-analysis pooling the results of eleven prospective cohort studies reported a significant association between elevated levels of CRP and IL-6 and subsequent depressive symptoms in a sample of 18,527 adults^28^. Another study systematically reviewed the evidence from six prospective cohorts studies investigating the inflammation hypothesis of depressive symptomatology in adults aged 60 years and older and found mixed results^22^. Five of these studies reported significant associations between interleukin 1 receptor antagonist, IL-6, IL-8 or TNF-α and future depressive symptoms^54–58^. However, Stewart et al. found that neither raised levels of fibrinogen nor CRP predicted subsequent depressive symptoms in a sample of American older adults^59^. This is in line with recent evidence from two large longitudinal cohort studies using data from the ELSA^29,60^. Heterogeneity in findings may be due to differences in study designs, population characteristics, and operationalisations of constructs. Another explanation may be the lack of regard for persistent depressive symptoms (i.e., persistent versus transient depressive symptoms)^31^ and the effects of repeated exposure to inflammation^61^. Furthermore, most studies have not considered plausible differential effects of inflammation on symptom-specific dimensions of depression, potentially masking the effects inflammation exerts on depressive symptoms. Our findings corroborate with the work of a recent study by Iob et al. (2019), which found that systemic low-grade inflammation exhibited stronger associations with somatic symptoms compared with cognitive-affective symptoms of depression^31^. Likewise, experimental administration of pro-inflammatory cytokines (e.g., TNF- α) or cytokine inducers (i.e., endotoxins) has been shown to initiate primarily somatic symptoms in both healthy participants^62–65^ and animal studies^66^. Together, these findings suggest that systemic inflammation may be a primary driver of somatic symptoms. Such evidence underlines the importance of considering conceivable symptom-specific profiles of depression in future studies. This may facilitate the development of more targeted treatment approaches.

This study has a number of strengths, including the prospective study design, the large sample size, and the statistical methods employed to investigate the link between PGS for depressive symptoms, systemic low-grade inflammation and specific symptom dimensions of depression. However, our findings need to be interpreted in light of several limitations. First, the measure of systemic low-grade inflammation was based on a single biomarker, which may not be sufficiently precise to understand the complexity of inflammatory processes in depression. Second, although we employed the most suitable factor analytic structure for the CES-D to distinguish between cognitive-affective and somatic symptom dimensions^50^, there are inconsistencies in the literature regarding the conceptualisation of somatic symptoms^67^. Last, the generalisability of the present findings is limited to Caucasian older adults only, since the ELSA population is largely comprised of white participants.

Future studies are needed to replicate our findings and further extend the present analyses to symptom-specific clusters of other depression scales, including a wider range of cognitive-affective and somatic items. Moreover, it is worth investigating the potentially mediating effects of multiple inflammatory markers, such as IL-6, fibrinogen, and TNF-α. In addition, further work is required to investigate intermediate mechanisms linking DS-PGS with cognitive-affective symptoms. It is also possible that systemic low-grade inflammation acts as a moderator in the relationship between DS-PGS and depressive symptoms. Therefore, longitudinal mediator-moderator models are needed to shed further light on the role of inflammatory processes. Lastly, research should explore the mediating effect of systemic inflammation through studying the relationship between PGS for systemic inflammation and subsequent depressive symptoms.

In conclusion, the current study demonstrated that polygenic susceptibility to depressive symptoms was significantly associated with both cognitive-affective and somatic symptoms of depression in a large population-based cohort of English older adults. Furthermore, systemic low-grade inflammation mediated the relationship between DS-PGS and somatic symptoms, but not the association between DS-PGS and cognitive-affective symptoms. This highlights the importance of investigating the independent effects of both PGS and inflammation on symptom-specific typologies of depression in future studies. Ultimately, this will provide further information about pathophysiological mechanisms of depression and advance the design of more targeted treatment approaches.

## Supplementary information

Supplementary Material

## Data Availability

The ELSA data containing the individual items and the derived scores for each of the respondents have been made available via UK Data Service (https://discover.ukdataservice.ac.uk).

## Code availability

The Mplus codes used for these analyses can be made available upon request.
