# Supplementary material for: Genetic susceptibility, inflammation and specific types of depressive symptoms: evidence from the English Longitudinal Study of Ageing
Source: Transl Psychiatry. 2020 May 12;10:140. doi: 10.1038/s41398-020-0815-9 (PMC7217832; doi:10.1038/s41398-020-0815-9)
Supplement: Supplementary file 1 — Supplementary Material [file 41398_2020_815_MOESM1_ESM.docx]

**SUPPLEMENTARY ANALYSES**

Figure S1. Confirmatory Factor Analysis (CFA) of the CES-D 8: One-factor versus two-factor measurement model of depressive symptomatology (wave 8, 2016/17) (N = 4 609)

Figure S2. Longitudinal mediation model of the association between polygenic scores for depressive symptomatology, C-reactive protein (wave 6, 2012/13) and specific types of depressive symptoms (wave 8, 2016/17), additionally adjusted for cognitive-affective and somatic symptoms (wave 6) (N = 3 510)

**Figure S1**: Confirmatory Factor Analysis (CFA) of the CES-D 8: One-factor versus two-factor measurement model of depressive symptomatology (wave 8, 2016/17) (N = 4 609)


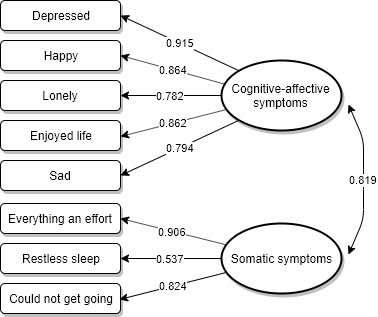

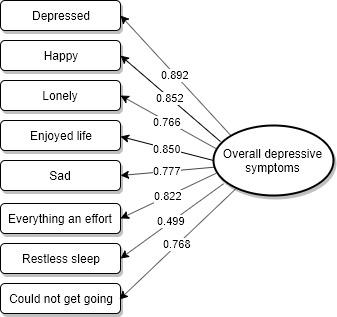


Model fit information: CFI = 0.969: RMSA = 0.069 Model fit information: CFI = 0.985; RMSA = 0.049

N = 4 609 N = 4 609

**Figure S2**: Longitudinal mediation model of the association between polygenic scores for depressive symptomatology, C-reactive protein (wave 6, 2012/13) and specific types of depressive symptoms (wave 8, 2016/17), additionally adjusted for cognitive-affective and somatic symptoms (wave 6) (N = 3 510)


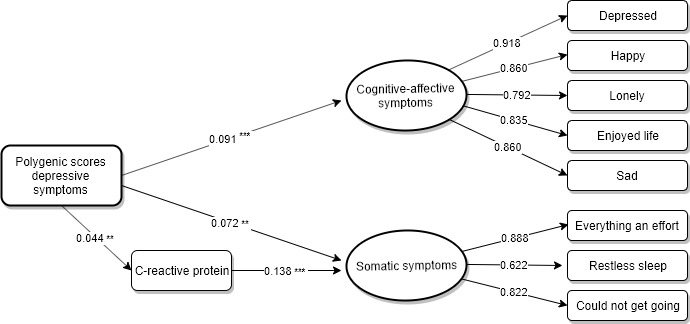


Data source: ELSA, waves 2-8: Estimator: WLSMV. Model adjusted for age, sex, 10 principal components, and cognitive-affective and somatic symptoms at wave 6.

p-value significance level: * < 0.05, ** < 0.01, *** < 0.001
